# *Caenorhabditis elegans*: a model to investigate oxidative stress and metal dyshomeostasis in Parkinson's disease

**DOI:** 10.3389/fnagi.2014.00089

**Published:** 2014-05-19

**Authors:** Patricia M. Chege, Gawain McColl

**Affiliations:** The Florey Institute of Neuroscience and Mental Health, University of MelbourneParkville, VIC, Australia

**Keywords:** *C. elegans*, oxidative stress, metals, Parkinson's disease, α-synuclein, tau, microtubules, axonal transport

## Abstract

Parkinson's disease (PD) is characterized by progressive motor impairment attributed to progressive loss of dopaminergic (DAergic) neurons in the *substantia nigra pars compacta*. Additional clinical manifestations include non-motor symptoms such as insomnia, depression, psychosis, and cognitive impairment. PD patients with mild cognitive impairment have an increased risk of developing dementia. The affected brain regions also show perturbed metal ion levels, primarily iron. These observations have led to speculation that metal ion dyshomeostasis plays a key role in the neuronal death of this disease. However, the mechanisms underlying this metal-associated neurodegeneration have yet to be completely elucidated. Mammalian models have traditionally been used to investigate PD pathogenesis. However, alternate animal models are also being adopted, bringing to bear their respective experimental advantage. The nematode, *Caenorhabditis elegans*, is one such system that has well-developed genetics, is amenable to transgenesis and has relatively low associated experimental costs. *C. elegans* has a well characterized neuronal network that includes a simple DAergic system. In this review we will discuss mechanisms thought to underlie PD and the use of *C. elegans* to investigate these processes.

## Introduction

Parkinson's disease (PD) is the second most prevalent age-related neurodegenerative disorder of the central nervous system, after Alzheimer's disease (AD). Idiopathic or sporadic PD affects approximately 1% of people over 65 years old (Hirtz et al., 2007). PD is characterized by severe motor impairment, which is attributed to profound depletion of striatal dopamine (DA) due to progressive loss of dopaminergic (DAergic) neurons in the *substantia nigra pars compacta*, a region in the basal ganglia that is crucial in voluntary motor functions (Hornykiewicz and Kish, 1987; Wooten, 1997; Braak et al., 2003). PD is also characterized by proteinaceous neuronal inclusions known as Lewy bodies (Irizarry et al., 1998). Current PD therapies focus mainly on correcting this DA depletion. Although effective in alleviating symptoms, these treatments lose their efficacy over time and do not halt the underlying neurodegeneration (Smith et al., 2012). Determining the mechanisms contributing to PD neurodegeneration is critical to facilitate the design of effective therapies to halt further neuronal loss.

While some PD cases are monogenic, arising from single point mutation in a specific gene, more than 90% of the cases are idiopathic (Table 1). The mechanisms underlying idiopathic PD are not fully understood. However, increasing evidence suggests that oxidative stress may be a major contributing factor to neuronal loss. This is evidenced by increased levels of oxidized lipids, proteins and nucleic acids in PD brains (Dexter et al., 1989a, 1994; Jenner and Olanow, 1996; Yoritaka et al., 1996; Alam et al., 1997a,b). Oxidative stress is thought to arise from a variety of mechanisms including mitochondrial dysfunction, neuroinflammation, perturbed DA metabolism and environmental toxins (Thomas and Beal, 2007; Hwang, 2013). Metal ion dyshomeostasis has also been hypothesized to cause oxidative stress, following evidence that PD brains exhibit increased total iron concentration (Dexter et al., 1991; Gotz et al., 2004; Oakley et al., 2007). In addition, levels of zinc are increased and copper decreased in the *substantia nigra* (Dexter et al., 1991).

**Table 1:** **PD associated and susceptibility genes and corresponding *C. elegans* homologs**.

***PARK* designation^a^**	**PD-associated genes**			
	**Gene**	**Type of mutation**	**Status**	***C. elegans* homolog**
*PARK1*	*SNCA*	Dominant	Confirmed	No known homolog
*PARK2*	*Parkin*	Recessive	Confirmed	*pdr-1*
*PARK3*	Unknown	Dominant	Not validated since first publication	Unknown
*PARK5*	*UCHL-1*	Dominant or risk factor	Unconfirmed; conflicting reports (Healy et al., 2006)	*ubh-1*
*PARK6*	*PINK1*	Recessive	Confirmed	*pink-1*
*PARK7*	*DJ-1*	Recessive	Confirmed	*djr-1.1 and drj-1.2*
*PARK8*	*LRRK2*	Dominant	Confirmed	*lrk-1*
*PARK9*	*ATP13A2*	Recessive	Confirmed	*catp-6*
*PARK11*	*GIGYF2*	Dominant	Unconfirmed; conflicting reports (Pankratz et al., 2002; Bras et al., 2009; Tan et al., 2009)	No known homolog
*PARK12*	Unknown	Risk factor	Confirmed	Unknown
*PARK13*	*HTRA2*	Dominant or risk factor	Unconfirmed; conflicting reports (Strauss et al., 2005; Simon-Sanchez and Singleton, 2008)	No known homolog
*PARK14*	*PLA2G6*	Recessive	Confirmed	Potential homologs: *C45B2.6, D1037.5, F47A4.5, H23L24.2, T04B2.5*, and *W07A8.2*
*PARK15*	*FBXO7*	Recessive	Confirmed	No known homolog
*PARK16*	Unknown	Risk factor	Confirmed	Unknown
*PARK17*	*VPS35*	Dominant	Confirmed	*vps-35*
*PARK18*	*EIF4G1*	Dominant	Not validated since first publication (Chartier-Harlin et al., 2011)	*ifg-1*
*PARK19*	*DNAJC6*	Recessive	Recently published (Edvardson et al., 2012; Koroglu et al., 2013)	*dnj-25*
*PARK20*	*SYNJ1*	Recessive	Recently published (Krebs et al., 2013; Quadri et al., 2013)	*unc-26*
	**PD susceptibility genes^b^**			
**Gene**	**Protein**		***C. elegans* homolog**	
*MAPT*	Tau		*ptl-1*	
*GBA*	Beta-glucosidase		*gba-1, gba-2, gba-3*, and *gba-4*	
*MC1R*	Melanocyte-stimulating hormone receptor		No known homolog	
*ADH1C*	Alcohol dehydrogenase 1C		*H24K24.3* and *Y50D4C.2*	
*HLA locus*	Major histocompatibility complex		No known homolog	

a PARK designation represents genes that are putatively linked to PD in chronological order of their identification.

b Certain polymorphisms or mutations in these genes pose a risk factor for PD.

Investigating the molecular basis of neurodegeneration *in vivo* relies on animal models, with mammalian models typically being used. All animal models have inherent experimental limitations and none fully replicate all aspects of a disease such as PD. As greater understanding of PD is gained and new hypotheses proposed there is a parallel need for animal models to be updated and modified to further our understanding. Establishing new transgenic models can have a significant lead-time with some animal systems being less suited to genetic modification. These particular limitations can be alleviated by use of a less complex animal, such as *Caenorhabditis elegans.*

## *C. elegans* as a neurodegeneration model

**Figure 1:**
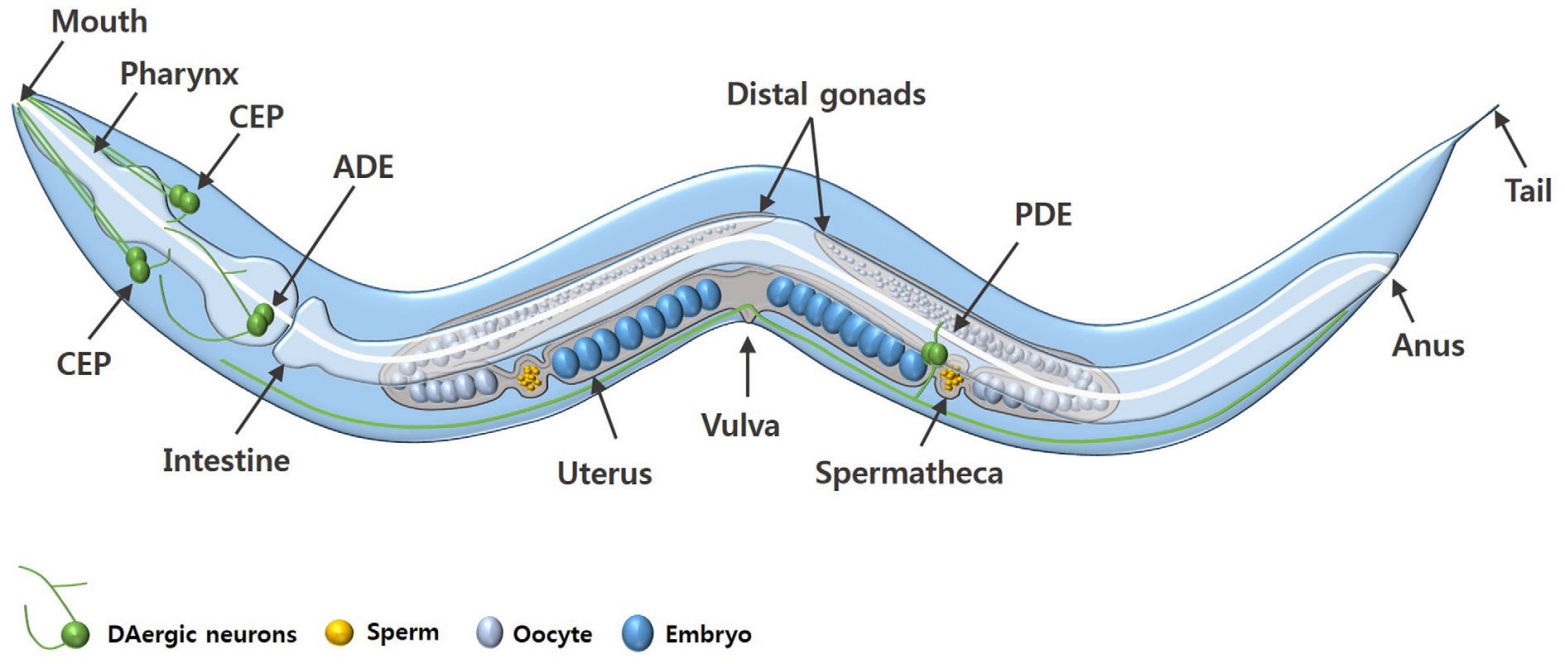
**An adult *C. elegans* hermaphrodite**. The diagram shows the key anatomical features and the DAergic neurons (green) of *C. elegans*. The DAergic neurons include four cephalic (CEP) neurons, two anterior deirid (ADE) neurons, and two posterior deirid (PDE) neurons. Males have six additional DAergic neurons located in the tail (not shown).

*C. elegans* is a free-living nematode, approximately 1 mm in length, which exists as either a self-fertilizing hermaphrodite or as a male (Figure 1). *C. elegans* can be cultured inexpensively on an *E. coli* lawn on agar media and has a short defined life cycle (Brenner, 1974). The rapid life cycle coupled with a high reproductive capacity makes *C. elegans* a suitable tool for mutagenesis and compound screening approaches. *C. elegans* also has an adult lifespan of approximately 3 weeks and is an established model of biological aging. Additionally, the *C. elegans* genome has been fully sequenced which has revealed that about 80% of *C. elegans* genes have human homologs and at least 42% of human disease-related genes have a *C. elegans* homolog (Consortium, 1998; Culetto and Sattelle, 2000; Lai et al., 2000). Functional studies of corresponding or related human genes can be done via mutation (where available) or RNA interference (RNAi) (Fire et al., 1998; Hamamichi et al., 2008; Ruan et al., 2010).

**Figure 2:**
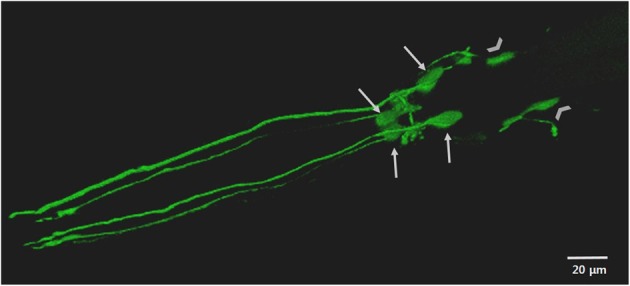
**The anterior DAergic neurons of an adult *C. elegans* hermaphrodite**. The neurons are visualized by the translational expression of GFP driven by the promoter of the DA transporter (P_dat-1_::GFP). The key features highlighted include the cell bodies and dendritic processes of the four CEP neurons (arrows) and the cell bodies of the two ADE (chevrons).

To complement these approaches or in the absence of endogenous homologs, *C. elegans* can be transgenically manipulated to express human disease associated genes in specific cell types, including neurons (Faber et al., 1999; Lakso et al., 2003; Brandt et al., 2009; McColl et al., 2009, 2012). Adult hermaphrodite *C. elegans* have 302 neurons, a neuronal network that is stereotypical between animals and which possesses most of the major neurotransmitter systems found in mammals, including DAergic neurons (White et al., 1986; Rand and Nonet, 1997; Bargmann, 1998). *C. elegans* are also optically transparent, which in conjunction with fluorescent protein reporters, allows for *in vivo* visualization of neurons, such as in Figure 2 (Chalfie et al., 1994; Nass et al., 2002; Chew et al., 2013).

As with other animal models, use of *C. elegans* to model disease is always tempered by an awareness of the limitations of cellular and anatomical differences. For example, *C. elegans* lack a vascular system and the somatic tissues of adult *C. elegans* are post-mitotic. Despite these obvious differences, key discoveries in *C. elegans* have been readily translated to vertebrate research. *C. elegans* was used to identify genes that are involved in regulating programmed cell death (Hedgecock et al., 1983; Ellis and Horvitz, 1986). dsRNA gene expression regulation was characterized in *C. elegans* and led to development of RNAi, a tool widely used in functional genomics (Fire et al., 1998). Additionally, the conserved effects of the insulin/insulin growth factor-1 signaling pathway on longevity were first noted in *C. elegans* mutants (Friedman and Johnson, 1988; Kenyon et al., 1993; Dorman et al., 1995; Murakami and Johnson, 1996; Kimura et al., 1997). *C. elegans* research has also linked iron metabolism to restless leg syndrome (Catoire et al., 2011). The findings in *C. elegans* were predictive of the role of ferritin in human tissue.

## Parkinson's disease

The hallmark PD symptoms are motor deficits, which include resting tremor, rigidity, slowness in movement (bradykinesia) and posture instability. In the majority of PD cases, these clinical manifestations only appear when approximately 50–70% of nigral neurons are lost and approximately 80% of striatal DA is depleted (Hornykiewicz and Kish, 1987; Kish et al., 1988; Orth and Schapira, 2002). This DA deficiency leads to the observed motor impairments because DA is an essential motor control neurotransmitter. In addition to DAergic degeneration, extensive neurodegeneration and atrophy occurs in other nerve cell types and brain regions as PD advances. The regions affected include the hippocampus, thalamus, and neocortex. This additional neurodegeneration leads to non-motor symptoms that include insomnia, depression, psychosis and cognitive impairment (Braak et al., 2003; Weintraub et al., 2011; Smith et al., 2012). These non-motor symptoms worsen over time, for example, an estimated 80% of PD patients with mild cognitive impairment develop dementia (Janvin et al., 2006; Buter et al., 2008; Hely et al., 2008). The etiology of the neurodegeneration leading to cognitive impairment remains unclear.

PD is also characterized by neuronal inclusions, Lewy bodies and Lewy neurites, which mainly contain aggregated α-synuclein (Forno, 1996; Spillantini et al., 1997; Irizarry et al., 1998). Alpha-synuclein is a 140-amino acid peptide encoded by the *SNCA* gene and is predominantly located at presynaptic terminals. It is highly expressed in the *substantia nigra*, hippocampus, neocortex, thalamus and cerebellum (Ueda et al., 1993; Nakajo et al., 1994; Iwai et al., 1995; Recchia et al., 2004). These brain regions are highly impacted by neurodegeneration in PD pathology. Several heritable point mutations, A30P, A53T, E46K, H50Q, and G51D, and a triplication of the *SNCA* gene are implicated in autosomal dominant forms of familial PD (Polymeropoulos et al., 1997; Kruger et al., 1998; Singleton et al., 2003; Zarranz et al., 2004; Appel-Cresswell et al., 2013; Proukakis et al., 2013). These findings have initiated numerous studies into the involvement of α-synuclein in idiopathic PD pathology.

Although several possibilities have been proposed, the function of α-synuclein remains unknown. Alpha-synuclein KO mice have impaired spatial learning and working memory suggesting some involvement in cognitive function (Kokhan et al., 2012). Sequestration of the protein in Lewy bodies may contribute to cognitive impairment seen in advanced PD. Alpha-synuclein over-expression in transgenic mice inhibits DA synaptic release while α-synuclein deficiency causes decreased vesicle-bound striatal DA (Abeliovich et al., 2000; Nemani et al., 2010). This suggests that α-synuclein is involved in synaptic transmission by regulating DA release. Alpha-synuclein deficiency may lead to unregulated DA release, which when coupled with loss of DAergic neurons, leads to the striatal DA depletion observed in PD. Under normal physiological conditions, α-synuclein negatively modulates the dopamine transporter (DAT), which is required for re-uptake of synaptically released DA (Wersinger and Sidhu, 2003). This implies that α-synuclein deficiency caused by sequestration in Lewy bodies may lead to increased DA re-uptake, causing increased concentration of intracellular DA. High levels of unbound intracellular DA have been shown to be neurotoxic (Olanow and Arendash, 1994; Luo et al., 1998; Offen et al., 1999; Lee et al., 2001).

Other studies suggest that α-synuclein may be a microtubule-associated protein (MAP) as it interacts with tubulin (Alim et al., 2002, 2004), with α-synuclein deficiency postulated to lead to microtubule dysfunction. Microtubules provide structural scaffolding in neurons and so their dysfunction would compromise neuronal integrity leading to neuron death. Alpha-synuclein sequestration in Lewy bodies appears to have significant implications in PD pathology, potentially by inhibiting the normal functions of α-synuclein, which may include facilitating cognitive function, synaptic transmission and stabilizing neuronal morphology. However, the underlying mechanisms that trigger DAergic neuronal death and α-synuclein aggregation in idiopathic PD require further investigation.

### *C. elegans* and DAergic neurons

*C. elegans* hermaphrodites have a comparatively simple DAergic system comprising eight neurons in total: six anterior DAergic neurons, which include four cephalic (CEP) neurons and two anterior deirid (ADE) neurons, and two posterior deirid (PDE) neurons (Figures 1, 2). Males have six additional DAergic neurons located in the tail (Sulston et al., 1975). DA synthesis, storage and transport mechanisms are conserved in *C. elegans* and DAergic nerve endings and synaptic vesicles have DA levels similar to those in mammalian neurons (Fuxe and Jonsson, 1973; Bargmann, 1998).

The functions of DAergic neurons have been investigated using laser ablation, a technique which can target a specific neuron while leaving neighboring neurons intact. The loss of DAergic neurons revealed that they are important for food searching and the basal slowing response upon sensing food (Sawin et al., 2000; Hills et al., 2004). Exposure to exogenous DA resulted in decreased egg laying, slowed defecation and paralysis (Schafer and Kenyon, 1995; Weinshenker et al., 1995; Hills et al., 2004; McDonald et al., 2006). Studies of mutations in *cat-2*, the tyrosine hydroxylase which is the rate limiting enzyme in DA synthesis, showed loss of basal slowing response and decreased touch habituation suggesting that DA signaling is necessary for mechanosensation (Sawin et al., 2000; Sanyal et al., 2004). These findings suggest that DAergic neurons are important for locomotion, associative learning, food searching, food sensing, egg-laying and defecation.

Most models of DAergic neurodegeneration in *C. elegans* are induced through exposure to neurotoxins and some metals, which selectively ablate DAergic neurons. These toxins include 6-hydroxydopamine (6-OHDA), l-methyl-4-phenylpyridinium (MPP+), methylmercury (MeHg), and manganese (Table 2) (Nass et al., 2002; Braungart et al., 2004; Settivari et al., 2009; VanDuyn et al., 2010). When exposed to 6-OHDA, *C. elegans* show a progressive and selective DAergic neuron degeneration and loss as evidenced by formation of blebs in axonal and dendritic membranes. (Nass et al., 2002; VanDuyn et al., 2010).

**Table 2:** ***C. elegans* Parkinson's disease models**.

**Gene**	**Construct/allele name^a^**	**Expression pattern**	**Phenotype**	**References**
α-*synuclein (human wild type)*	*P_*dat*-1_::α-synuclein*	DAergic neurons	DAergic neurodegeneration, motor deficits, reduced DA and α-synuclein accumulation in DAergic neurons	Lakso et al., 2003; Kuwahara et al., 2006; Cao et al., 2005
	*P_*aex*-3_::α-synuclein*	Pan-neuronal	DAergic neurodegeneration	Lakso et al., 2003
	*P_*unc*-51_::α-synuclein*		Endocytosis, motor and developmental defects	Kuwahara et al., 2008
	*P_*snb*-1_::α-synuclein*		Mitochondrial stress	Ved et al., 2005
	*P_*unc*-54_::α-synuclein::GFP*	Body wall muscles	α-synuclein accumulation	Hamamichi et al., 2008
	*P_*unc*-54_::α-synuclein::YFP*		α-synuclein accumulation	van Ham et al., 2008
	*P_*acr*-2_::α-synuclein*	Motor neurons	Reduced motor movements	Lakso et al., 2003
	*P_*mec*-7_::α-synuclein*	Touch-receptor neurons	Impaired touch sensitivity	Kuwahara et al., 2008
α-*synuclein (human mutant)*	*P_*dat*-1_::α-synuclein (A30P), (A53T), (A56P)*, and *(A76P)*	DAergic neurons	DAergic neurodegeneration	Karpinar et al., 2009
	*P_*dat*-1_::α-synuclein (A53T)*		DAergic neurodegeneration	Lakso et al., 2003
	*P_*dat*-1_::α-synuclein (A30P)* and *(A53T)*		Reduced DA and α-synuclein accumulation in DAergic neurons	Kuwahara et al., 2006
	*P_*unc*-51_::α-synuclein (A53T)* and *(A30P)*	Pan-neuronal	Endocytosis, motor and developmental defects	Kuwahara et al., 2008
	*P_*unc*-119_::α-synuclein (A53T)*		Mitochondrial stress	Ved et al., 2005
	*P_*aex*-3_::α-synuclein (A53T)*		DAergic neurodegeneration, motor deficits	Lakso et al., 2003
	*P_*acr*-2_::α-synuclein (A53T)*	Motor neurons	Reduced motor movements	Lakso et al., 2003
	*P_*mec*-7_::α-synuclein (A53T)*	Touch-receptor neurons	Impaired touch sensitivity	Kuwahara et al., 2008
*GFP*	*P_*dat*-1_::GFP*	DAergic neurons	Visualizes the DAergic neurons	Nass et al., 2002
*MAPT (human tau)*	*P_*aex*-3_::tau (WT)*	Pan-neuronal	Uncoordinated movement	Kraemer et al., 2003
	*P_*aex*-3_::tau (V337M)*		Insoluble tau accumulation	
	*P_*aex*-3_::tau (P301L)*		Nerve cord degeneration	
*LRRK2*	*P_*snb*-1_::LRRK2 (WT)*	Pan-neuronal	Mitochondrial stress	Saha et al., 2009
	*P_*snb*-1_::LRRK2 (R1441C)*		Mitochondrial stress	
	*P_*snb*-1_::LRRK2 (G2019S)*		Mitochondrial stress, DAergic neurodegeneration and reduced DA levels	
*Protein with tau like repeats (ptl-1)*	*ok621*	Null mutant	Early on-set neurodegeneration, egg hatching defects and reduced touch sensitivity	Gordon et al., 2008; Chew et al., 2013
	*tm543*	Partial deletion mutant	Early on-set neurodegeneration	Chew et al., 2013
**Chemical treatment**	**Phenotype**			**References**
*6-hydroxydopamine (6-OHDA)*	DAergic neurodegeneration			Nass et al., 2002; Cao et al., 2005
*MPTP/MPP+*	DAergic neurodegeneration			Braungart et al., 2004; Pu and Le, 2008
*Methyl mercury (MeHg)*	DAergic neurodegeneration			VanDuyn et al., 2010
*Manganese*	DAergic neurodegeneration and oxidative stress			Settivari et al., 2009
*Aluminum*	DAergic neurodegeneration			VanDuyn et al., 2013

a Construct name includes the promoter used to drive the transgene (promoter::transgene).

### *C. elegans* and α-synuclein

Although *C. elegans* lacks a human α-synuclein homolog, α-synuclein expression has been investigated in transgenic *C. elegans*. The targeting of transgene expression in *C. elegans* body wall muscle cells has been used to explore the toxicity of several disease-associated proteins. Body wall muscles run longitudinally along the length of the nematode and are essential for locomotion. Functional disruption of these cells causes a clear and robust paralysis phenotype (McColl et al., 2009, 2012). In PD research, green or yellow fluorescent protein-tagged α-synuclein was expressed in the body-wall muscle of *C. elegans* to visualize α-synuclein aggregation *in vivo* (Hamamichi et al., 2008; van Ham et al., 2008). These lines have been used to screen RNAi libraries, revealing 20 neuroprotective genes whose knock down enhanced α-synuclein aggregation. One of these genes was the ortholog of human VSP41, a key lysosomal trafficking protein that protects against toxicity of DA-derived neurotoxins (Hamamichi et al., 2008; Ruan et al., 2010). Another genome-wide RNAi screen revealed 80 genes that when knocked down accelerated formation of α-synuclein inclusions. These genes, which appear to suppress inclusion formation, are predominantly involved in vesicular transport and lipid metabolism (van Ham et al., 2008). These findings suggest that defects in the endosomal-lysosomal and ER-Golgi vesicular trafficking system pathways may be implicated in α-synuclein toxicity.

Additionally, neurodegenerative processes can also be studied directly in *C. elegans* neurons. Over-expression of wild type and A53T mutant α-synuclein under the control of pan-neuronal promoter, *aex-3* and under the DAergic neuron specific promoter, *dat-1*, caused loss of DAergic neurons (Lakso et al., 2003; Cao et al., 2005). Two neuroprotective endoplasmic reticulum (ER) associated proteins, TorsinA and Rab1 A, were found to ameliorate α-synuclein toxicity and prevent neuron loss (Cao et al., 2005; Cooper et al., 2006), suggesting that α-synuclein toxicity affects the ER-Golgi vesicular trafficking system. Another model over-expressing wild type or mutant α-synuclein under the control of the pan-neuronal promoter, *unc-51*, was used to screen an RNAi library for genetic modifiers that either suppress or exacerbate α-synuclein toxicity. Knock down of four genes that are involved in synaptic endocytosis enhanced α-synuclein toxicity (Kuwahara et al., 2008), suggesting that impaired endocytosis may contribute to α-synuclein dysfunction seen in PD pathology.

Wild type and A53T mutant (human) α-synuclein have been transgenically over-expressed via the *C. elegans* DAergic neuron specific promoter, *dat-1* (Lakso et al., 2003; Cao et al., 2005). A screen of 115,000 compounds in cells and then *C. elegans* identified four 1,2,3,4-tetrahydroquinolinones antagonists of α-synuclein toxicity (Su et al., 2010). Another larger screen revealed that several 8-hydroxyquinolines could ameliorate α-synuclein aggregation and toxicity in *C. elegans* (Tardiff et al., 2012). The underlying mechanism of protection is proposed to be via interplay between metal homeostasis and proteotoxicity of aggregation prone proteins. Interestingly another 8-hydroxyquinoline, PBT2, has been found to reduce (the Alzheimer's associated peptide) Aβ toxicity in transgenic *C. elegans* (McColl et al., 2012). This compound is currently under clinical trial as an AD therapeutic (Lannfelt et al., 2008; Crouch et al., 2011).

## Oxidative stress and metal homeostasis

Oxidative stress occurs from an imbalance between toxic oxidant production and antioxidant activity, which leads to cellular damage followed by apoptosis (Sies, 1991; Jenner, 2003). The main reactive oxidants are the reactive oxygen species (ROS) and the reactive nitrogen species (RNS). RNS have been comprehensively reviewed elsewhere (Jomova et al., 2010). ROS, such as superoxide (O^•−^_2_) and hydroxyl radical (^•^OH) are normal by-products of oxygen consumption during cellular metabolism, predominantly in the mitochondria (Kepp, 2012). ROS levels are tightly regulated by endogenous antioxidant enzymes, such as glutathione, superoxidase dismutase (SOD), and catalase (Bains and Shaw, 1997; Sohal and Orr, 2012). It is important to stress that ROS have essential functions in normal cell biology and are not always inherently detrimental. For example, ROS are a component of the innate immune system, particularly in phagocytes, which produce ROS to prevent colonization by microbes (Fang, 2004). ROS are also utilized in cellular signaling (Hekimi et al., 2011) to modulate the activity of kinases, phosphatases and transcription factors. However, ROS are detrimental when their production goes unchecked leading to damage of cellular lipids, proteins and nucleic acids, and ultimately cell death (Pattison et al., 2002; Niki, 2009).

A way to counter the detrimental effects of ROS overproduction could be to administer antioxidant supplements or drugs, such as, Vitamins A, C, and E and compounds that inhibit ROS production. However, antioxidant therapeutic interventions have not been successful in alleviating oxidative stress associated with neurodegenerative diseases. This is primarily due to the inability of these compounds to effectively cross the blood brain barrier (Halliwell, 2001). Additionally, these antioxidants when administered in high doses have negative side effects by affecting normal cellular processes that rely on ROS activity (Halliwell, 2001; Freeman and Keller, 2012). An understanding of the cause of oxidative stress is vital to design better therapies to prevent neurodegeneration.

Biological transition metals, such as iron, copper, zinc, magnesium, nickel, cobalt, and manganese, are essential co-factors for at least one-third to one-half of all proteins (Andreini et al., 2008; Waldron et al., 2009). Iron and copper are metabolically utilized due to their ability to redox cycle, with iron being the most abundant. However, in the event of metal ion misregulation, this redox ability has the potential to produce toxic radicals via Haber-Weiss and Fenton reactions leading to oxidative stress (Nunez et al., 2012). Levels of these metal ions are reported to be perturbed in brains affected by various neurodegenerative diseases. This has led to the metal ion dyshomeostasis hypothesis, which proposes that the metal ion imbalance triggers increased ROS production causing oxidative stress that eventually leads to neuronal death. It is plausible that the observed metal imbalance is just a symptom and not a cause of neurodegeneration. However, several heritable neurodegenerative diseases are directly caused by metal-ion misregulation. These progressive conditions include aceruloplasminaemia and neuroferritinopathy, which result from iron misregulation, and Menkes Disease and Wilson's Disease, which result from copper misregulation (Vulpe et al., 1993; Yoshida et al., 1995; Harris et al., 1998; Curtis et al., 2001). These diseases suggest that dyshomeostasis of brain metals is sufficient to initiate neurodegeneration.

Iron is an essential metal in organisms because of its redox ability (Cairo et al., 2002). For example, reactive iron is part of the cytochrome complex in the mitochondrial respiration chain, which is important for cellular energy production. It is a crucial co-factor for catalase, an antioxidant that regulates hydrogen peroxide levels and also for heme proteins, which are essential for vascular transport of oxygen and carbon-dioxide. In the *substantia nigra*, iron is essential for DA synthesis (Youdim et al., 1984). However, this reactivity also allows iron to catalyze production of toxic hydroxyl radicals via Fenton chemistry:

$$\begin{array}{l} {\text{Fe}}^{3+}+{\text{H}}_{2}{\text{O}}_{2}\to {\text{Fe}}^{2+}+{\text{HOO}}^{•}+{\text{H}}^{+}\\ {\text{Fe}}^{2+}+{\text{H}}_{2}{\text{O}}_{2}\to {\text{Fe}}^{3+}+{\text{OH}}^{-}+{\;}^{•}\text{OH} \end{array}$$

Therefore, the concentration of unbound intracellular iron must be kept low; a process regulated by iron storage and transport proteins, such as ferritin (iron storage), ferroportin (iron efflux), divalent metal transporter-1 (DMT-1, an iron transporter), and transferrin (iron shuttling/uptake) (Lee and Andersen, 2010; Gkouvatsos et al., 2012). Disruption in these homeostatic functions could result in iron accumulation leading to oxidative damage and loss of function of proteins that depend on iron as a co-factor. This could potentially disrupt cellular respiration, antioxidant activity, oxygen/carbon dioxide transport and DA synthesis.

Copper is an important co-factor in the activity of redox active proteins, such as ceruloplasmin (iron homeostasis), cytochrome c oxidase (mitochondrial cellular respiration), Cu/Zn-superoxide dismutase (antioxidant activity) and dopamine-b-hydroxylase and tyrosinase, which are key proteins in DA synthesis (Arredondo and Nunez, 2005; Kepp, 2012). Therefore, copper imbalance in neurons may affect the function of these proteins. Additionally, unbound copper concentration requires tight control due to its redox potential. Copper levels higher than 10^−18^ M can initiate oxidative damage by facilitating ROS production (Rae et al., 1999):

$${\text{Cu}}^{+}+{\text{H}}_{2}{\text{O}}_{2}\to {\text{Cu}}^{2+}+{\text{OH}}^{-}+{\;}^{•}\text{OH}$$

Copper levels are predominantly regulated by ion importers, copper efflux pumps (ATP7A and ATP7B), metallochaperones, metalloregulators and other copper regulating proteins, such as, ceruloplasmin (Cp), (Camakaris et al., 1999; Waldron et al., 2009; Pang et al., 2013). Defects in these systems may result in increased levels of unbound copper causing oxidative damage. In addition, copper misregulation may cause loss of function of the copper dependent proteins, with resultant negative implications on iron homeostasis, cellular energy metabolism, oxidative stress responses and DA synthesis.

### Parkinson's disease and oxidative stress

PD brains show increased levels of oxidized macromolecules, which can be used as an indirect measure of ROS levels. Malondialdehyde, lipid hydroperoxides and 4-hydroxynonenal, which are lipid peroxidation products, are increased in PD brains (Dexter et al., 1989a, 1994; Yoritaka et al., 1996). PD brains also show increased levels of 8-hydroxydeoxyguanosine (8-OHdG) and protein carbonyls, which are products of DNA and protein oxidation, respectively, (Alam et al., 1997a,b). Another marker of elevated ROS levels in PD brains is increased SOD activity in the *substantia nigra* (Marttila et al., 1988; Saggu et al., 1989). SOD catalyzes the dismutation of superoxide (O^•−^_2_) into oxygen and hydrogen peroxide, therefore its activity may increase as a neuroprotective measure to cope with increased ROS levels.

Increased ROS levels not only lead to cellular damage but also to production of oxidation by-products that are also potentially neurotoxic. For example, 4-hydroxynonenal irreversibly modifies α-synuclein aggregation *in vitro*, potentially leading to formation of protofibrils, which are neurotoxic to cultured DAergic neurons (Qin et al., 2007). Lipid hydroperoxides have been shown to lead to oxidation of DA to 6-OHDA, a known neurotoxin (Sauer and Oertel, 1994; Przedborski et al., 1995; Pezzella et al., 1997; Lotharius and O'Malley, 2000). Additionally, α-synuclein aggregation can be induced *in vitro* in the presence of hydrogen peroxide (Hashimoto et al., 1999). This suggests that increased ROS levels not only directly cause neuronal damage but also indirectly contribute to DA depletion and α-synuclein aggregation, which can further exacerbate PD progression.

Taken together, these findings suggest that PD brains are under oxidative stress, which leads to neurodegeneration. However, the mechanisms underlying the increase in ROS levels are not clearly understood. Mitochondrial dysfunction, neuroinflammation, DA autoxidation and environmental toxins have been implicated in the increase of ROS in PD brains (Thomas and Beal, 2007; Jomova et al., 2010; Hwang, 2013). Metal ion dyshomeostasis may also lead to increased ROS production in PD. Generally, the *substantia nigra* has the highest distribution of iron in the central nervous system. However, PD brains have more elevated levels of iron in this region (Dexter et al., 1989b; Riederer et al., 1989; Sofic et al., 1991; Good et al., 1992; Gerlach et al., 1994; Vymazal et al., 1999; Haacke et al., 2007). The infusion of iron into rat brains results in parkinsonism and behavioral changes (Ben-Shachar and Youdim, 1991; Sengstock et al., 1993). Additionally, in mice the 8-hydroxyquinoline metal ion chelator, clioquinol, and over-expression of ferritin, an iron storage protein, both prevent neurodegeneration in PD models (Kaur et al., 2003). These findings suggest that iron may play a significant role in PD neurodegeneration.

The elevated iron levels in the *substantia nigra* are proposed to directly and indirectly contribute to increased ROS production. Increased unbound iron levels can produce ROS, such as superoxide, via Fenton chemistry (Halliwell and Gutteridge, 1986). Additionally, ferric ions can precipitate oxidation of DA to 6-OHDA in the presence of hydrogen peroxide (Pezzella et al., 1997). Superoxide and 6-OHDA have the ability to release iron stored in ferritin and [4Fe-4S] cluster-containing enzymes (Liochev and Fridovich, 1994). This can potentially lead to a vicious cycle in which unbound iron increases levels of superoxide and 6-OHDA causing release of more unbound iron. This may contribute to the progressive neurodegeneration observed in PD.

Iron dyshomeostasis not only contributes to ROS production but also negatively impacts the function of proteins that use iron as a co-factor. For example, tyrosine hydroxylase, the rate-limiting enzyme in DA synthesis, depends on iron (Nagatsu, 1995; Ponting, 2001). Therefore, an increase in iron as seen in PD brains may increase DA synthesis, causing excess DA to be released into the cytoplasm, which may lead to increased ROS production. This iron-induced DA dysfunction not only inhibits the normal function of DA but may also lead to increased DA oxidation into the neurotoxin 6-OHDA (Pezzella et al., 1997; Jiang et al., 2013).

In addition to increased ROS production and iron dyshomeostasis, PD brains also exhibit a reduction in metal ion storage capacity and antioxidant activity. Ferritin is a key iron storage protein and disruption of its function perturbs iron homeostasis. PD brains have decreased ferritin levels (Dexter et al., 1991). This potentially leads to iron storage deficiency, which allows unbound reactive iron to accumulate in the *substantia nigra*, facilitating ROS production (White and Munro, 1988; Dexter et al., 1991; Connor et al., 1995). Neuroferritinopathy is a condition caused by a genetic mutation of the ferritin light chain which disrupts ferritin assembly, leading to iron accumulation and neurodegeneration in the basal ganglia, resulting in severe motor disorders (Curtis et al., 2001; Vidal et al., 2003). In addition to decreased ferritin levels, PD patients have decreased concentration and activity of Cp (Kristinsson et al., 2012). Cp is a multi-copper oxidase that oxidizes ferrous ions (Fe^2+^) to less reactive ferric ions (Fe^3+^). This oxidation is essential for cellular iron uptake and efflux by ferroportin and transferrin. Aceruloplasminaemia, a heritable condition resulting from Cp deficiency, leads to iron accumulation in the basal ganglia, neurodegeneration and motor problems including dystonia and tremors (Harris et al., 1998).

PD brains have approximately 40% lower reduced glutathione (GSH), an antioxidant enzyme that catalyzes the reduction of ROS (Sofic et al., 1992). GSH also forms complexes with other enzymes, such as glutathione peroxidase and glutathione S-tranferases, to facilitate ROS reduction (Smeyne and Smeyne, 2013). Decreased antioxidant capacity likely contributes to the oxidative stress seen in PD brains. These findings suggest that defective metal ion transport and storage, decreased antioxidant activity and increased reactive metal ion accumulation contribute to oxidative stress leading to neurodegeneration.

### *C. elegans*: oxidative stress and metal ion hypothesis

Another link between PD and oxidative stress is gleaned from studies of mutations in *DJ-1* and *PINK1*, which are associated with early onset PD (Bonifati et al., 2003; Valente et al., 2004). *DJ-1* and PINK1 have been shown to protect against oxidative stress (Junn et al., 2005; Pridgeon et al., 2007). This was confirmed in *C. elegans* by studying the nematode homologs, *djr1.1* and *pink-1*. The *djr-1.1* knock-down and *pink-1* mutant strains showed increased sensitivity to toxin-induced oxidative stress (Ved et al., 2005; Samann et al., 2009). These *C. elegans* models complement the familial PD studies and strengthen the hypothesis that oxidative stress contributes to PD pathology.

Transgenic *C. elegans* expressing α-synuclein in neurons exhibited mitochondrial fragmentation attributed to α-synuclein interaction with mitochondrial membranes, affecting membrane fusion (Kamp et al., 2010). Alpha-synuclein is localized in mitochondria, suggesting that α-synuclein dysfunction potentially contributes to mitochondrial dysfunction in PD (Li et al., 2007). In turn, mitochondrial dysfunction leads to ROS overproduction in the *substantia nigra* of PD brains, which leads to cellular damage and cell death.

A recent study using electron paramagnetic resonance demonstrated that unbound reactive iron levels increased during oxidative stress in *C. elegans* (Rangel et al., 2012). Increased iron levels in *C. elegans* resulted in increased protein oxidation, suggesting that iron triggers increased ROS production. Iron chelation using deferoxamine and over-expression of ferritin (*ftn-1*) reduced protein oxidation (Valentini et al., 2012). Knock down or deletion of *C. elegans* SMF-1/2/3 (orthologs of human iron transporter, DMT-1) partially inhibits DAergic neuronal death (Settivari et al., 2009; VanDuyn et al., 2013). PD brains have increased iron levels, decreased ferritin levels and increased DMT-1 levels, consistent with defective iron transport and storage systems in PD brains (Dexter et al., 1991; Salazar et al., 2008).

Knock down of SKN-1 (a *C. elegans* ortholog of Nrf2), a transcription factor that regulates expression of glutathione S-transferase, increased susceptibility to metal-induced neurodegeneration in DAergic neurons (VanDuyn et al., 2010; Settivari et al., 2013). This finding and the observation that PD brains show decreased levels of glutathione suggests that decreased antioxidant activity contributes to PD pathology (Sofic et al., 1992).

Metal dyshomeostasis and oxidative stress may represent an important component underlying idiopathic PD. *C. elegans* possesses homologs of some of the iron homeostasis proteins (Table 3) therefore the nematode can be used to further our understanding of metal homeostasis in relation to PD. Even more broadly, any findings can potentially be extended to familial autosomal PD because *C. elegans* also has homologs for the majority of genes implicated in familial PD (Table 1). These features may be exploited to investigate these genes and their interactions with metal homeostasis.

**Table 3:** ***C. elegans* iron metal homeostasis proteins**.

**Human protein**	***C. elegans* homolog**
Ferritin	Ferritin 1 (FTN-1)
	Ferritin 2 (FTN-2)
Ceruloplasmin	F21D5.3
Ferroportin	Ferroportin 1.1 (FPN-1.1)
	Ferroportin 1.2 (FPN-1.2)
	Ferroportin 1.3 (FPN-1.3)
Divalent metal-ion transporter	SMF-1
	SMF-2
	SMF-3

## The missing link

**Figure 3:**
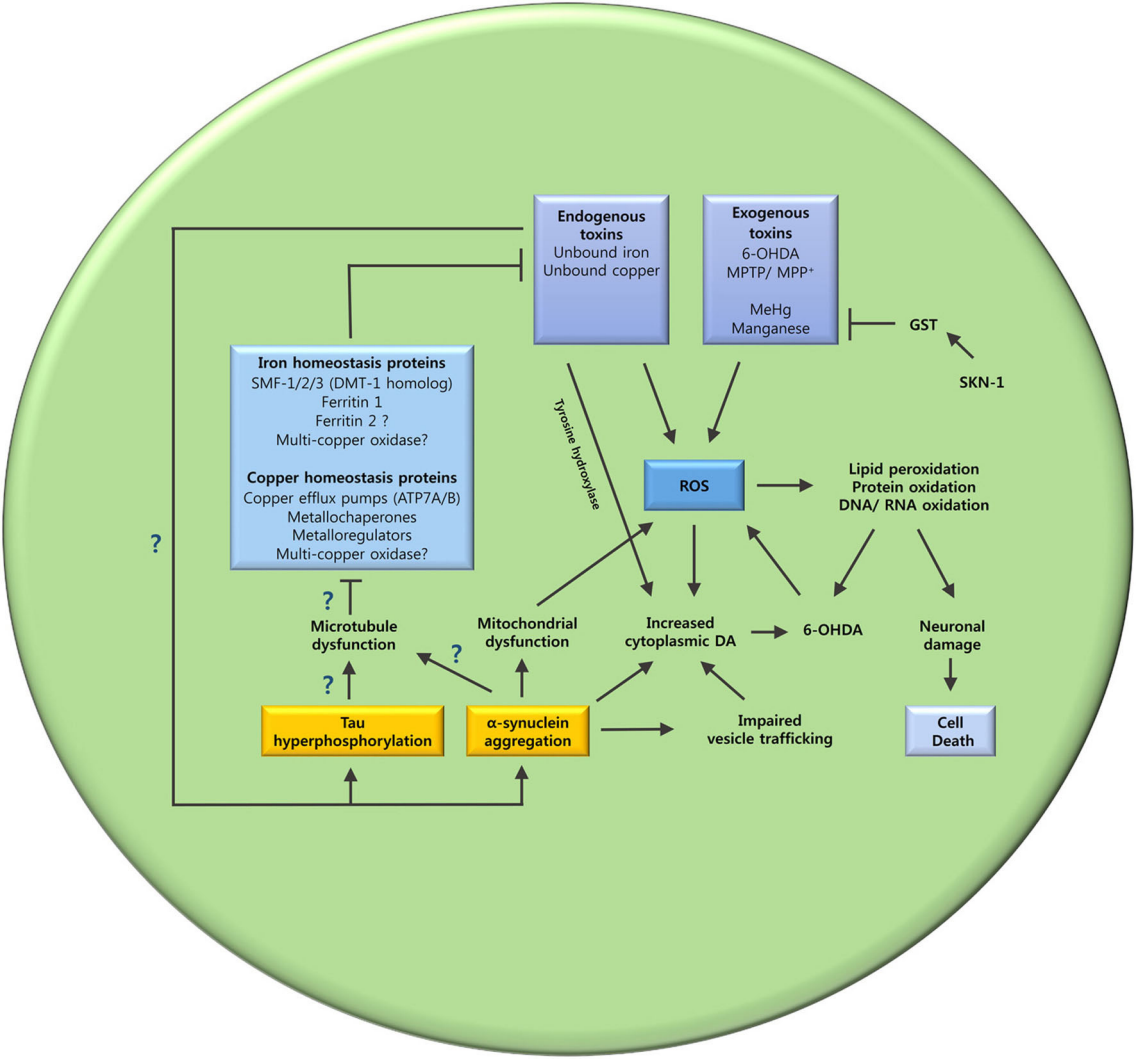
**The oxidative stress and metal ion dyshomeostasis cascade**. This schematic summarizes the hypothesized mechanisms that may lead to oxidative stress and metal dyshomeostasis in DAergic neurons of *C. elegans* PD models. The unanswered questions are also highlighted. Does tau hyperphosphorylation and α-synuclein aggregation cause microtubule dysfunction? Are key metal ion homeostasis proteins dependent on microtubules? Does metal ion dyshomeostasis contribute to tau hyperphosphorylation and α-synuclein aggregation? GST (glutathione S-transferase); SKN-1 (Nrf2 ortholog).

Varied evidence supports the potential role of metal dyshomeostasis in PD neurodegeneration. However, the underlying mechanism that leads to metal imbalance still remains to be elucidated. The interplay between tau, α-synuclein and microtubules, may hold an answer to this question (Figure 3).

### Tau, α-synuclein, and parkinson's disease

Tau is a MAP predominantly expressed in axons and is thought to regulate the assembly of microtubules (Weingarten et al., 1975; Kosik and Finch, 1987). Neurofibrillary tangles (NFTs) comprised of hyperphosphorylated tau aggregates are a pathological hallmark of AD (Kidd, 1963; Wischik et al., 1988). Although not often emphasized, tau has also been implicated in PD pathology. Some PD patients have NFTs and in older people with parkinsonian symptoms, the severity of gait impairment appears to correlate with the degree of NFT accumulation (Joachim et al., 1987; Bancher et al., 1993; Schneider et al., 2006). Certain single-nucleotide polymorphisms in the tau gene pose an increased risk factor for PD (Zabetian et al., 2007; Edwards et al., 2010). Tau KO mice have recently been reported to exhibit neuronal iron accumulation, *substantia nigra* neuronal loss, parkinsonism and cognitive deficits (Lei et al., 2012). Anti-psychotic DA D2 receptor antagonists, such as azaperone, suppress insoluble tau aggregation in *C. elegans* (McCormick et al., 2013), suggesting an interplay between tau and DA.

Increasing evidence highlights the importance of tau and α-synuclein in PD pathology and indicates that the two proteins significantly interact. Tau is co-localized with α-synuclein in Lewy bodies (Arima et al., 1999). Tau and α-synuclein can seed and promote each other's polymerization to form insoluble aggregates (Giasson et al., 2003; Geddes, 2005). Alpha-synuclein has been shown to directly facilitate tau phosphorylation and also to mediate glycogen synthase kinase 3 (GSK-3β, a serine/threonine protein kinase) catalyzed tau phosphorylation, which is increased in PD brains (Jensen et al., 1999; Muntane et al., 2008; Duka et al., 2009). This indicates that α-synuclein may contribute to the increased GSK-3β activity, which leads to tau hyperphosphorylation.

### Tau, α-synuclein, and microtubule dysfunction

Based on the interaction between tau and α-synuclein, the dysfunction of the two proteins may disrupt two key functions of microtubules: axonal transport and maintaining neuronal morphology. Microtubule dysfunction precedes impaired axonal transport (Cartelli et al., 2013). This was deduced from altered mitochondria distribution and neurodegeneration in DAergic neurons of mice exposed to MPTP. MPTP is known to destabilize microtubules and impair axonal transport specifically in DAergic neurons (Cappelletti et al., 2005; Ren et al., 2005; Morfini et al., 2007). Administration of a microtubule stabilizer, Epothilone D, attenuated further nigrostriatal neurodegeneration (Cartelli et al., 2013), highlighting a potential link between axonal transport disruption, microtubule dysfunction and neurodegeneration.

As a MAP, tau not only stabilizes microtubules but also regulates transport by serving as a physical barrier and by interacting with transport motor proteins, dynein and kinesin, to regulate microtubule attachment and detachment (Jancsik et al., 1996; Trinczek et al., 1999; Stamer et al., 2002; Mandelkow et al., 2003; Magnani et al., 2007; Dixit et al., 2008). Tau over-expression disrupts the transport of mitochondria and vesicles leading to accumulation of mitochondria in distal parts of the neuron (Ebneth et al., 1998; Stamer et al., 2002; Mandelkow et al., 2003). Hyperphosphorylated tau filaments have been shown to phosphorylate the kinesin light chain thereby triggering the dissociation of kinesin from its cargo (Lapointe et al., 2009). Phosphorylation of tau at the amino terminus can also impact its inhibitory effect on axonal transport (Kanaan et al., 2012). Mutant tau has been shown to cause “traffic jams” which inhibit axonal transport (Shemesh et al., 2008). The tau dysfunction observed in PD may negatively impact axonal transport, contributing to neurodegeneration.

Alpha-synuclein is co-localized with tubulin in Lewy bodies and co-purifies with microtubules. Additionally, when incubated with tubulin, α-synuclein polymerizes tubulin into microtubules (Alim et al., 2002, 2004). Immunofluorescence staining of α-synuclein transfected COS-1 cells with α-synuclein and tubulin antibodies, showed that α-synuclein co-localized predominantly with microtubules (Alim et al., 2004). Alpha-synuclein binds synaptic vesicles via its amino terminus and is involved in vesicle trafficking (Jensen et al., 1999; Cooper et al., 2006). These findings suggest that α-synuclein, like tau, is a MAP and is involved in axonal transport of vesicles. Alpha-synuclein dysfunction likely leads to impaired axonal transport.

Protein with tau-like repeats (PTL-1) is the only known tau/MAP2 ortholog in *C. elegans* (Goedert et al., 1996). PTL-1 is important for maintaining *C. elegans* neuronal morphology (Chew et al., 2013). Null mutants for *ptl-1* show accelerated neurite branching and microtubule bundle disorganization in mechanosensory and GABAergic neurons (Chew et al., 2013). Microtubule changes in these neurons suggest a link between tau deficiency and compromised neuronal integrity. In addition, transfection of *ptl-1* into non-neuronal cells promotes microtubule assembly and bundling (Goedert et al., 1996).

Loss of function of tau and α-synuclein may result in significant microtubule disruption that leads to neurodegeneration seen in PD. Although the downstream effect of microtubule dysfunction in neurons remains to be elucidated, we can speculate that cellular functions which rely upon microtubules will be disrupted. The function of metal ion regulating proteins, such as ferroportin and copper transporter (ATP7A), are dependent on axonal transport (Cobbold et al., 2004; Moos and Rosengren Nielsen, 2006). Microtubule disruption would likely lead to disrupted trafficking of these metal ion homeostasis proteins. This in turn disrupts metal ion homeostasis leading to accumulation of unbound reactive metal ions and metal ion deficiency, resulting in oxidative stress followed by neuronal loss.

## Conclusion

Despite evidence pointing to the involvement of metal ion imbalance and microtubule dysfunction in neurodegeneration, few studies have attempted to link these two elements. We propose that disrupted axonal transport and neuronal integrity greatly impacts metal ion balance by hindering the trafficking of metal ion homeostasis proteins and neuronal anti-oxidants (Figure 3). Disrupting metal ion homeostasis is likely to result in oxidative stress leading to neuronal loss. In addition, microtubule disruption may result in loss of synaptic connections due to altered neuronal morphology causing synaptic transmission impairment. The interplay between tau, α-synuclein and metal dyshomeostasis offers a new avenue of investigation. *C. elegans* has homologs for many of the genes involved in iron regulation (Table 3) and can be genetically manipulated to express transgenes in the absence of homologs (Table 2); this may represent an ideal system in which to investigate these questions.

### Conflict of interest statement

The authors declare that the research was conducted in the absence of any commercial or financial relationships that could be construed as a potential conflict of interest.
